# Retinal nerve fibre layer loss in hereditary spastic paraplegias is restricted to complex phenotypes

**DOI:** 10.1186/1471-2377-12-143

**Published:** 2012-11-23

**Authors:** Sarah Wiethoff, Ahmad Zhour, Ludger Schöls, Manuel Dominik Fischer

**Affiliations:** 1 Department of Neurology and Hertie-Institute for Clinical Brain Research, University of Tübingen, Tübingen, Germany; 2 German Center of Neurodegenerative Diseases (DZNE), Tübingen, Germany; 3 University Eye Clinic, Centre for Ophthalmology, Tuebingen, Germany; 4 Hertie-Institute for Clinical Brain Research, Otfried-Müller Str. 27, 72076, Tübingen, Germany

**Keywords:** Hereditary spastic paraplegia (HSP), Spastic paraplegia rating scale (SPRS), Eye, Retinal nerve fibres, Optical coherence tomography (OCT)

## Abstract

**Background:**

Reduction of retinal nerve fibre layer (RNFL) thickness was shown as part of the neurodegenerative process in a range of different neurodegenerative pathologies including Alzheimer′s disease (AD), idiopathic Parkinson’s disease (PD), spinocerebellar ataxia (SCA) and multiple system atrophy (MSA). To further clarify the specificity of RNFL thinning as a potential marker of neurodegenerative diseases we investigated RNFL thickness in Hereditary Spastic Paraplegia (HSP), an axonal, length-dependent neurodegenerative pathology of the upper motor neurons.

**Methods:**

Spectral domain optical coherence tomography (OCT) was performed in 28 HSP patients (clinically: pure HSP = 22, complicated HSP = 6; genetic subtypes: SPG4 = 13, SPG5 = 1, SPG7 = 3, genetically unclassified: 11) to quantify peripapillary RNFL thickness. Standardized examination assessed duration of disease, dependency on assistive walking aids and severity of symptoms quantified with Spastic Paraplegia Rating Scale (SPRS).

**Results:**

HSP patients demonstrated no significant thinning of global RNFL (*p*_global_ = 0.61). Subgroup analysis revealed significant reduction in temporal and temporal inferior sectors for patients with complex (p<0.05) but not pure HSP phenotypes. Two of three SPG7-patients showed severe temporal and temporal inferior RNFL loss. Disease duration, age and severity of symptoms were not significantly correlated with global RNFL thickness.

**Conclusion:**

Clinically pure HSP patients feature no significant reduction in RNFL, whereas complex phenotypes display an abnormal thinning of temporal and temporal inferior RNFL. Our data indicate that RNFL thinning does not occur unspecifically in all neurodegenerative diseases but is in HSP restricted to subtypes with multisystemic degeneration.

## Background

Hereditary spastic paraplegias (HSPs) are a group of genetically determined progressive neurodegenerative disorders where dysfunction of axonal transport processes predominantly leads to a length-dependent degeneration of corticospinal tract fibres [1]. They are clinically classified as “pure” or “uncomplicated” HSPs when the pathology is limited to the core symptom of progressive lower limb weaknesses and spasticity, optionally accompanied by neurogenic bladder disturbance and mild diminution of lower-extremity vibration sense. “Complicated” or “complex” HSPs additionally show different system involvement including cerebellar affection, lower motor neuron involvement, sensory impairment, peripheral neuropathy, dementia, mental retardation and more rarely deafness, psychiatric disorders, seizures, extrapyramidal signs or extraneurological signs (e.g. ichthyosis) [2-4]. Ophthalmologic affection like congenital cataract, optic atrophy or retinitis pigmentosa has not been systematically studied but is only rarely reported [5].

HSPs are genetically heterogenous and can be inherited as autosomal dominant, autosomal recessive, or X-linked recessive trait. So far at least 48 chromosomal loci are associated with HSP referred to as spastic paraplegia genes (SPG) SPG1-SPG48 [6]. The most common subtype is SPG4 with an autosomal-dominant inheritance and a predominantly pure phenotype [7].

The retinal ganglion cells deliver a unique study object with their unmyelinated axons and direct synapses into the central nervous system. Optical coherence tomography is a highly suitable method to study the thickness of retinal nerve fibre layer in vivo *and* non-invasively. There is clear evidence for RNFL thinning in multiple sclerosis (MS) with and without optic neuritis as well as in neurodegenerative pathologies like Multiple System Atrophy (MSA), Alzheimer disease (AD), Parkinson’s disease (PD) or degenerative ataxias [8-13]. Yet, to our knowledge by submission, no study has been performed to investigate RNFL in HSP patients which makes the study an important addendum in order to gain more insight into the pathology of HSP and address the question, whether RNFL thinning represents a rather nonspecific affection in all neurodegenerative disorders, or whether it might retain some specificity. HSP is a perfect model to address this question as it is thought to derive from distal axonopathy e.g. due to axonal transport defects. HSP preferentially affects the longest axons of the corticospinal tracts declining to the legs and spares shorter axons like those innervating the arms. Retinal axons comprising the RNFL are comparably short and thus would be expected to remain spared in case RNFL reduction is not an unspecific marker of neurodegenerative per se. To investigate possible RNFL reduction in HSP patients, we designed a prospective observational study as our observations e.g. in MSA patients with significant RNFL thinning confirm, that these changes might very well remain subclinical and not likely to be detected in routine clinical examination [Fischer et al., unpublished data].

## Methods

### Subjects

We prospectively examined 31 HSP patients, 7 of complex and 24 of pure phenotype (HSP-c and HSP-p, respectively). Diagnosis of HSP was made according to Harding and GeNeMove criteria [2,14]. Inclusion criteria included (distant) best corrected visual acuity (D-BCVA) of < 0.3 logarithm of the minimum angle of resolution (logMAR), spherical ametropia of ≤ ± 4 dpt. and cylindrical ametropia ≤ ± 2 dpt. Exclusion criteria comprised the existence of other neurodegenerative diseases or ocular pathology that could falsify test results, e.g. glaucomatous neuropathies which led to a final study population of 28 patients.

Disease causing mutations were known for 17 of 28 patients including 13 patients with *SPAST* mutation (SPG4; all with clinically pure phenotypes), 3 with *paraplegin* mutations (SPG7; two clinically complex, one pure), 1 with *CYP7B1* mutations (SPG5 with pure phenotype). The control group consisted of 28 healthy subjects matched for gender with mean age of 38 years (SD: 10,3) who met the same ophtalmological inclusion and exclusion criteria and did not show any sign of spastic paraparesis or other neurological disorder.

The independent ethics committee of the University of Tuebingen approved the study, which was performed in accordance to the ethical standards of the Declaration of Helsinki (1964). All patients gave written informed consent prior to their inclusion in the study.

### Retinal Imaging

An initial complete ophthalmologic exam was performed to exclude confounding neuroophthalmological disorders such as glaucoma. Spectral domain optical coherence tomography (SD-OCT) imaging and analysis was performed using the Spectralis HRA + OCT platform (Heidelberg Engineering, Heidelberg, Germany) with real time compensation for eye movements as described elsewhere [9]. Briefly, for quantification of central retinal thickness (CRT) and RNFL thickness, we used the proprietary Eye Explorer software (version 5.1), which automatically defines RNFL thickness as distance between inner limiting membrane (ILM) and ganglion cell layer (GCL) and CRT as distance between ILM and retinal pigment epithelium. For acquisition of circular scans, a mean of 16 images was calculated with automated alignment of iterative recordings using the automated real-time mode, thereby increasing the signal-to-noise ratio by a factor of four [15]. CRT was measured from transverse sections (also using averaged recordings of 16 images) through the foveal center in an orthogonal fashion (cross hair) to exclude oblique measurements due to tilt. Scans with poor quality (< 20 dB) were excluded from the analysis.

### Neurological examination

Age of onset was defined as the onset of a progressive gait disorder. Family history covered first and second degree relatives. Disease severity and complicating symptoms were assessed by the Spastic Paraplegia Rating Scale (SPRS) [14]. SPRS-scores range from 0–52 points with higher scores indicating more severe disease. Complicating symptoms are reflected in a clinical inventory. See Table 1 for mean age, disease duration, clinical features and SPRS scoring for all HSP patients as well as subgroups.

**Table 1:** Clinical data of the whole cohort as well as clinical and molecular-genetic subtypes

	**Age [years]**	**Disease duration [years]**	**SPRS [scoring points]**	**Phenotype**
**HSP-a (n=28)**	48.1 *(8–75)*	19.1 (*4*–*40)*	20.8 (*3*–*30)*	complex & pure, see subgroups
**HSP-p (n=22)**	49.5 *(8–75)*	21.8 *(5–43)*	21.5 *(3–31)*	spastic paraparesis, pure phenotype
**HSP-c (n=6)**	42.8 *(22–60)*	9.5 *(4–18)*	18 *(8–31)*	most frequent complicating symptoms: gaze-evoked-nystagmus or ataxia
**SPG4 (n=13)**	46.1 *(8–74)*	21.5 *(7–37)*	20.8 *(3–30)*	spastic paraparesis, pure phenotype
**SPG5 (n=1)**	48	43	23	pure phenotype
**SPG7 (n=3)**	38.3 *(28–48)*	9.7 *(4–14)*	17.7 *(8–31)*	one pure, two complex phenotypes (see text)

Data are given as mean values and range in brackets for all HSP-patients (HSP-a) and for clinically pure phenotypes (HSP-p), complex phenotypes (HSP-c) as well as molecular-genetic (SPG4, SPG5, SPG7) subgroups.

### Statistical analysis

All data are reported as mean value +/−standard deviation (SD) and a *p*-value of < 0.05 was regarded as statistically significant (data uncorrected for multiple comparisons). To assess whether a correlation exists between OCT-data and SPRS-scores, duration of disease or age, Pearson′s correlation test was used.

Analysis of variance (ANOVA) was used to determine a significant difference in foveal thickness and RNFL thickness sector data sets (JMP V8.02, SAS Institute Inc., Cary, USA).

## Results

Ophthalmologic examination showed signs of confounding ocular pathology in none of the healthy controls but in three of the patients. Two pure HSP patients with a mutation of the *SPG4 gene* and one patient with complex HSP displayed glaucomatous optic disc cupping with positive family history of primary open angle glaucoma in 2 of 3 patients. These patients were excluded from further analysis via OCT. One patient with SPG7 was diagnosed with optic atrophy but was not excluded from the analysis as optic atrophy was regarded as part of the multidegenerative process in complex HSP that is subject of this study.

### Spectral domain optical coherence tomography (SD-OCT)

Quality indices (signal to noise ratio [dB]) of OCT recordings in both groups were high (HSP: 29.96 ± 4.63, range 21.00 - 42.00; CTRL: 29.43 ± 3.05, range 24.00 - 38.00) indicating robust primary data sets.

#### Whole HSP-cohort

HSP patients demonstrated a tendency to reduced foveal thickness and thinning of overall RNFL, which both failed to reach statistical significance compared to the control group. Changes in RNFL did not describe a uniform pattern in HSP. Rather, temporal superior and nasal superior sectors showed tendencially increased RNFL thickness in patients compared to healthy controls, whereas a slight reduction in RNFL thickness in patients was observed in temporal, nasal, nasal inferior, and temporal inferior sectors. Overall, greatest change was observed in foveal total retinal thickness measurements of HSP patients.

#### Pure HSP

Patients with pure HSP did neither show any significant reduction of global RNFL nor thinning in any of the individual sectors. Only total retinal thickness at the fovea was significantly thinner in patients with pure HSP (p<0.05).

#### Complex HSP

In patients with complex forms of HSP (HSP-c) RNFL thickness was significantly reduced within temporal (p<0.05) and temporal inferior sections (p<0.05).

#### SPG4

All SPG4-patients displayed a clinically pure phenotype. No abnormalities of RNFL were observed apart from reduced foveal total retinal thickness (p<0.05).

#### SPG5

The SPG5-patient presented with a pure clinical phenotype and RNFL measurements within the normal range in all retinal sectors.

#### SPG7

Two of three SPG7 patients had complex phenotypes. One 39 year old with a disease duration of 4 years presented with ataxia and gaze-evoked nystagmus as complicating symptoms. The second patient was diagnosed with optic atrophy and displayed cognitive impairment, dysarthria and gaze-evoked nystagmus in addition to spastic paraplegia. The third SPG7-patient had pure spasticity of lower limbs with a disease duration of 11 years without complicating symptoms.

**Figure 1:**
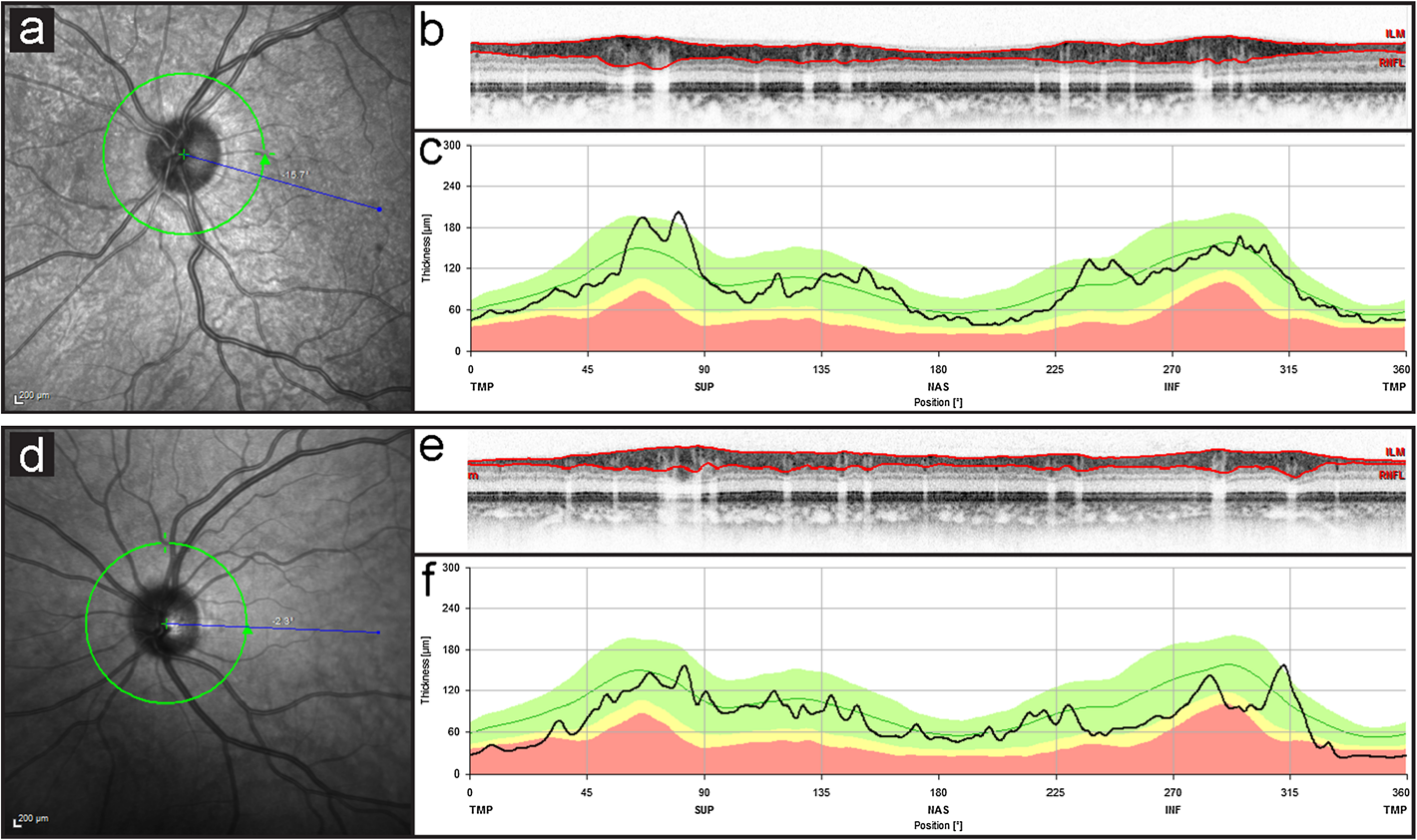
**Exemplary SD-OCT data sets in individual pure (top) and complex (bottom) HSP patients.** Left panels (**a**,**d**) demonstrate anatomy of the optic nerve head and indicate placement of the peripapillary ring scan (green circle) in relation to the point of fixation (blue line) recorded by simultaneous scanning laser ophthalmoscopy. In the patient with pure HSP (SPG4), the acquired virtual cross section (**b**) of the peripapillary retina demonstrates a normal RNFL thickness (delineated by the red lines). Quantitative spatial analysis (**c**) of this individuals RNFL thickness is displayed as black line in front of data from the normative data set provided by the software (green line = mean, green area = 95% confidence intervall, yellow area = 99% CI, red area = outside 99% CI). Conversely, recordings from a SPG7 patient with complex HSP (**d**-**f**) indicate severely decreased RNFL thickness. This was most pronounced in the temporal sector (43 μm, norm: 81 μm). Both temporal inferior (113 μm, norm: 152 μm) and nasal inferior (72 μm, norm: 109 μm) sectors were borderline to pathologic levels, whereas nasal (66 μm, norm: 72 μm), nasal superior (97 μm, norm: 102 μm) and temporal superior (121 μm, norm: 141 μm) sectors did not show significant reduction.

The two patients with complex phenotypes showed a global RNFL thickness outside normal limits, especially the temporal RNFL was decreased in both patients. Additionally, the nasal superior sector was borderline to pathology in one SPG7 patient and the nasal inferior sector in the other complex SPG7 patient. Nasal and temporal superior quadrants did not depict reduction of RNFL thickness (Figure 1). In the third SPG7-patient with a clinically pure phenotype no abnormalities in RNFL were detected. All RNFL measures are displayed in Table 2.

**Table 2:** Retinal nerve fibre layer (RNFL) thickness in different sectors

	**Global** (**μm)**	**Temporal** (**μm)**	**Temporal superior** (**μm)**	**Nasal superior** (**μm)**	**Nasal** (**μm)**	**Nasal inferior** (**μm)**	**Temporal inferior** (**μm)**	**Foveal** (**μm)**
**Controls (n=28)**	99.82±7.41	76.50±9.18	136.86±12.32	103.36±18.89	75.18±12.21	109.50±19.86	145.39±11.82	230.21±13.64
**HSP-a (n=28)**	98.57±10.67 p=0.61	71.86±14.23 p=0.15	138.39±17.19 p=0.70	104.71±16.74 p=0.77	75±14.16 p=0.96	107.57±19.27 p=0.71	143.54±20.23 p=0.68	223.61±15.5 p=0.1
**HSP-p (n=22)**	100.82±9.76 p=0.88	76.09±10.48 p=0.69	139.59±17.31 p=0.88	104.82±16.43 p=0.91	76.18±14.91 p=0.92	110.45±19.47 p=1	146.36±20.27 p=0.91	221.23±15.28 p*<0.05
**HSP-c (n=6)**	90.33±10.58 p=0.06	56.33±16.24 p*<0.05	134±17.58 p=0.66	104.33±19.48 p=0.82	70.67±10.93 p=0.92	97±15.54 p=0.68	133.17±17.88 p*<0.05	232.33±14.15 p=0.08
**SPG4 (n=13)**	103.77±10.43 p=0.98	77.31±11.53 p=0.57	141.23±18.19 p=1	108.92±18.55 p=0.91	78.77±15.33 p=0.91	115.69±22.29 p=0.54	151.92±21.98 p=0.83	219.01±13.31 p*<0.05
**SPG5 (n=1)**	98 p=n.a.	81 p=n.a.	139 p=n.a.	86 p=n.a.	72 p=n.a.	107 p=n.a.	143 p=n.a.	209 p=n.a.
**SPG7 (n=3)**	88.67±18.45 p=0.48	51.33±27.43 p=0.13	141±23.9 p=0.69	92.33±19.86 p=0.81	77±17.45 p=0.26	92.67±7.09 p=0.78	124.33±21.22 p=0.10	222±12.77 p=0.14

Data shown in μm as mean ± SD. Statistic comparison of subgroups was performed to the corresponding number of healthy controls. P-value as revealed by ANOVA if applicable (with restrictions to SPG7-subgroup (n=3) and SPG5-subgroup (n=1), p* = p < 0.05 (p uncorrected)). For abbreviations, see Table 1.

### Correlation with clinical features

#### Visual acuity

Our data set of visual acuity in complex and pure HSP patients was collected using standard ETDRS visual acuity tests and subgroup analysis showed no significant difference in visual acuity (HSP_complex_: 0.02 ± 0.08, range −0.10 - 0.10; HSP_pure_: -0.01 ± 0.07, range −0.10 - 0.10; p = 0.47). There was no significant difference regarding visual function in the HSP cohort compared to healthy controls (HSP: 0.00 ± 0.07, range −0.10 - 0.10; CTRL: -0.02 ± 0.07, range −0.10 - 0.10; p = 0.58). Furthermore, there was no significant correlation between RNFL thickness and visual function in these cohorts (r_HSP_ = 0.19; r_CTRL_ = 0.01).

#### Disease severity

There was no significant correlation between thickness of global RNFL or RNFL in different retinal sectors and disease severity quantified by SPRS score (r^2^_global RNFL versus severity_ = 0.05), disease duration (r^2^_global RNFL versus duration_ = 0.01) or age (r^2^_global RNFL versus age_ = 0.03) in the whole HSP-cohort. Within subgroups, correlation with clinical data did not reveal significant results.

## Discussion

Our study presents first data on RNFL measures in HSP patients in order to target the question whether RNFL loss can be observed unspecifically in any form of neurodegenerative disorder and whether the distribution pattern within the retina is specific for single disease entities as previously suggested [10,16]. Interestingly, some studies investigating RNFL in different neurodegenerative diseases claimed specific patterns of RNFL loss for the different disease entities even though there are still controversial findings to report on: E.g. in idiopathic Parkinson’s disease (PD), one study showed the RNFL to be significantly reduced in the (inferior-) temporal sector [10], other studies revealed statistically significant decrease of global RNFL [17] or reduced inner retinal thickness (RNFL, ganglion cell and inner plexiform layer) in both inferior and superior macular fields [16], while another study did not detect any statistically significant difference in measurements of retinal thickness at all [18]. According to the limited amount of published data available regarding RNFL in degenerative ataxias, patients with spinocerebellar ataxias type SCA1, SCA2 and SCA3 showed decreased RNFL thickness, whereas a reduced macular thickness was found in patients with SCA 1, 3 and 6 but not SCA2 [13,19]. OCT-measurements in MSA patients showed significant reduction of RNFL in nasal quadrants compared to healthy controls [9]. In contrast, patients suffering from Alzheimer’s disease featured significant RNFL decrease in the superior quadrant in one study [20] while all quadrants were reported affected in another study [12]. Final evidence of specific reduction patterns for each underlying neurodegenerative disorder has not yet been yielded, but most available data seem to confirm the notion that neurodegenerative/-inflammatory disorders such as AD, PD, MSA, some SCAs and MS are associated with a significant loss of retinal nerve fibres. While these observations have been gathered using different generations of OCT devices of varying axial resolution, signal to noise ratio and methods of artifact reduction, new studies from latest generation OCT devices further substantiate previous findings of significant morphological differences on a retinal level in patients with neurodegenerative/-inflammatory disorders [21,22].

In this study, HSP patients, in contrast to patients with other neurodegenerative disorders showed only a tendency towards global thinning of RNFL, but no statistically significant changes. RNFL thickness (global as well as within different sectors) did not correlate with clinical severity, duration of disease or age. This is in line with the current notion of molecular pathogenesis of HSP as a length dependent axonal degeneration, which widely restricts the degenerative process to neuronal fibre tracts with the longest axons e.g. corticospinal tracts, dorsal column and peripheral nerves of the lower limbs. Even though molecular mechanisms responsible for the assumed axonal degeneration in HSP are only partially understood, our findings fit into this concept. Here we show, that retinal ganglion cell axons forming the retinal nerve fibre layer are not affected by the degenerative process in pure HSP. This finding for the first time can bring up scientific data that discards the assumption of RNFL loss being an unspecific surrogate marker for all forms of neurodegeneration.

In contrast, patients with complex forms of HSP where the disease spreads beyond the long fibre tracts presented significant thinning of RNFL especially in temporal and temporal inferior sectors. These changes reached significance despite the small number of individuals with complex HSP included in this study. However, these changes were not statistically significant after correction for multiple comparisons and need to be reproduced in a larger cohort of patients with complex HSP. This may also help to depict genotype specific effects. So far we found patients with mutations in the nuclear-encoded mitochondrial AAA metalloproteinase paraplegin causing SPG7 [23] to present with retinal changes. Interestingly, within the small group of SPG7-patients abnormal thinning of RNFL was restricted to the two patients with complex phenotypes.

As mitochondrial defects are thought to play a crucial role in the pathogenesis of SPG7 and affection of retinal ganglion cells as well as optical atrophy are a common feature of other mitochondriopathies [5] it would be interesting to see whether thinning of the RNFL is a common feature in other mitochondriopathies. Our observation suggests that underlying mitochondrial pathology and a complex phenotype in HSP may predispose for developing optical atrophy. Further stratification of this risk potential is clearly needed in a bigger cohort of SPG7-patients but personal communication (Alexandra Dürr) suggests that RNFL is indeed frequently altered in SPG7. It would be of interest to learn whether patients with SPG31, another rare subform of HSP with mitochondrial dysfunction [24] also present RNFL loss like SPG7.

## Conclusions

As a conclusion, our study demonstrated that the RNFL is affected in the majority of HSP patients with complex phenotypes, but is not altered in SPG4 and pure forms of hereditary spastic paraplegia. This clearly shows that RNFL loss is not an unspecific change in any neurodegenerative disease but might emerge in certain subforms depending on the underlying molecular-genetic pathology and phenotype. One limiting factor concerning reliability of the data presented here definitively is the heterogeneity of the investigated cohort and the comparably small number of complex phenotypes studied. Therefore larger cohort studies will be needed to confirm the findings.

## Competing interests

The authors declare that they have no competing interests.

## Author’ s contributions

AZ assisted in ophthalmological assessments of patients and controls and overall study coordination. LS contributed substantially to the study design and to critical revisions of the manuscript. MDF carried out ophthalmological assessments including OCT-measurements, -analysis and figure-drafting and contributed substantially throughout the process of composing and submitting the manuscript. SW performed neurological examinations, statistical analysis of the data and wrote the manuscript. All authors read and approved the final manuscript.

## Pre-publication history

The pre-publication history for this paper can be accessed here:

http://www.biomedcentral.com/1471-2377/12/143/prepub
